# Subclinical ocular surface changes in children with newly diagnosed allergy

**DOI:** 10.3389/fped.2025.1648267

**Published:** 2025-10-10

**Authors:** Ata Baytaroğlu, Mesut Saka

**Affiliations:** ^1^ Ophthalmology Department, Hacettepe University, Ankara, Türkiye; ^2^ Pediatrics Department, Uşak University, Usak, Türkiye

**Keywords:** allergy, ocular surface, meibomian glands, dry eye, conjunctiva

## Abstract

**Objective:**

To investigate subclinical changes in the anterior segment of the eye in pediatric patients with newly diagnosed allergic rhinitis or dermatitis compared to healthy controls.

**Methods:**

This case-control study included children aged 3–15 years diagnosed with allergic rhinitis, dermatitis, or asthma. The control group consisted of healthy children matched for age and sex without systemic or ocular allergies. The Ocular Surface Disease Index (OSDI) questionnaire was administered with parental assistance, particularly to younger children. Ocular surface parameters, keratorefractive values, and corneal topographies were evaluated.

**Results:**

No significant differences were observed between the groups in terms of refractive status (*p* = 0.55). The OSDI scores were higher in the allergy group (*p* = 0.044), although the values remained below clinically relevant thresholds. Meibomian gland atrophy was significantly greater (24.4% vs. 15.8%, *p* = 0.008), and non-invasive tear break-up time (NiBUT) was significantly shorter (8.4s vs. 10.7s, *p* = 0.004) in the allergy group.

**Conclusion:**

Subclinical ocular surface changes, including increased meibomian gland atrophy and reduced tear stability, may occur in children with asymptomatic allergies. While these findings do not currently influence treatment, they highlight the need for longitudinal studies to evaluate the potential progression and inform future screening practices.

## Introduction

Atopy is a clinical syndrome characterized by a predisposition to IgE-mediated hypersensitivity responses to common allergens, which often results in allergic rhinitis, dermatitis, and asthma in children (1). While allergic conjunctivitis is a common comorbidity, it occurs in approximately 31% of children with systemic allergies, indicating that most pediatric patients with atopy do not exhibit obvious eye symptoms (2).

Emerging literature suggests that meibomian gland dysfunction (MGD) and tear film instability, which are common features of many ocular surface diseases, may also begin subclinically in pediatric allergic populations, even without concurrent conjunctivitis. In a study of the general pediatric population, Gupta et al. found that up to 42% of subjects showed signs of meibomian gland atrophy (3). This highlights that meibomian gland dysfunction (MGD) may begin early in life and increase throughout adolescence, with factors such as increased screening time, exposure to allergens, and recurring blepharoconjunctivitis (3–8).

The potential interplay between systemic allergy and subclinical ocular changes warrants further investigation to determine whether early markers of ocular surface disturbance warrant clinical attention (6, 9). Therefore, this study aimed to investigate whether children newly diagnosed with allergic rhinitis or dermatitis but without ocular complaints exhibit detectable ocular surface alterations.

## Method

### Participants

Patients aged >3 and <15 years who were diagnosed with allergic rhinitis, dermatitis, or asthma within the preceding week were included in the study. The inclusion criteria required a new clinical diagnosis of one of these allergies (within the past week) and no history of ocular allergic disease. All children were considered atopic by virtue of their diagnosed allergy, even if specific IgE tests were not performed, as non-IgE-mediated allergic pathways were also recognized as potential contributors to ocular changes. Although atopy is classically defined as an IgE-mediated response, non-IgE-mediated hypersensitivities or their exacerbations were included, because their associated systemic inflammatory pathways may also contribute to ocular surface alterations. The diagnoses of allergic rhinitis and asthma were based on comprehensive systemic examinations and tests in accordance with the Allergic Rhinitis and its Impact on Asthma (ARIA) 2016 and Global Initiative for Asthma (GINA)2024 guidelines. Due to the lack of definitive diagnostic criteria for allergic rhinitis, especially in preschool children, specific diagnostic measures, such as serum IgE or immediate hypersensitivity skin tests, were not required for all cases. No staging or subclassification was performed for allergic rhinitis or asthma. Dermatitis was diagnosed using the Hanifin-Rajka criteria (10). The exclusion criteria for both groups included refractive error exceeding 3 diopters, any previous ocular surgery, contact lens wear, regular screen time of more than two hours per day, any prior diagnosis of allergic keratoconjunctivitis, and any recent (within the past few weeks) respiratory tract infection. These criteria were implemented to minimize confounding factors and isolate the relationship between systemic allergy and ocular surface changes.

### Control cohort and matching

Healthy controls were recruited from the same pediatric clinic. For each case, the controls were matched at a 1:1 ratio. The matching criteria included age (within one year of birth) and sex. Controls were confirmed to have no history of systemic or ocular allergies through a parental questionnaire and a clinical review. The same exclusion criteria were used for the control group.

### Data collection

To avoid bias due to inter-eye correlation, only the right eye of each participant was evaluated, and the refractive status was determined as the spherical equivalent of the cycloplegic autorefractometry values. As part of the detailed anterior segment evaluation, central corneal thickness and keratometry values in diopters, horizontal white-to-white (WTW) measurements, and anterior chamber depth were recorded. In all cases, keratometric measurements were taken after 30 min of waiting, following two administrations of cyclopentolate 1% at 5 min intervals. In addition to the anterior segment, the evaluation included biomicroscopic assessment, OSDI, ocular staining with preservative-free fluorescein, and topographic dry eye analysis using Sirius+ (Costruzione Strumenti Oftalmici, Firenze, Italy) and Phoenix v.4.1.3.1. Topographic dry eye analysis was performed using Sirius+. While one researcher conducted all imaging procedures to ensure consistency, two researchers performed separate measurements from the recorded images using the device's tutorial to assess inter-observer agreement. Meibomian gland atrophy was quantified using Phoenix software embedded in the instrument. This software performs automated analysis of infrared meibography images of the upper and lower eyelids, calculating the percentage of gland loss (non-perfused area) relative to the total visible gland area. The reported value was the average of the upper and lower eyelids. The Ocular Surface Disease Index (OSDI; Allergan Inc., Irvine, CA, USA) questionnaire was administered with parental assistance to ensure the understanding of younger participants. While recognizing that the OSDI is not specifically validated for diagnosing or grading dry eye severity in children, it was used in this study as a standardized tool to screen for the presence of significant ocular surface symptoms, with parental input enhancing its reliability.

### Statistical analysis

The sample size was determined using G*Power Version 3.1.6 (Heinrich-Heine-Universität Düsseldorf, Germany). With an anticipated effect size of 0.5, approximately 50 participants per group were needed to achieve an alpha significance level of 0.05 and a power of 0.8. Statistical analyses were performed using SPSS 15.0 for Windows (SPSS Inc., Chicago, IL, USA). Ultimately, we enrolled 50 children in the allergy group and 50 in the control group, meeting the target sample size. Descriptive statistics are displayed as counts and percentages for categorical variables, and as means, standard deviations, minimums, maximums, and medians for numerical variables. When the normal distribution assumption was not met, numerical variables were compared using the Mann–Whitney *U* test for two independent groups. The group proportions were compared using the chi-squared test. Spearman correlation was used to analyze the relationships between numerical variables because the parametric test assumptions were not satisfied. To identify the factors associated with meibomian gland atrophy, separate multivariate linear regression analyses were performed for the allergy and control groups. This stratified approach was chosen to determine if the predictors of atrophy differed between healthy and allergic states. In each model, the percentage of meibomian gland atrophy was the dependent (outcome) variable. The independent (predictor) variables were limbal redness score, NiBUT, tear film layer thickness, and OSDI score. The significance level was set at *p* < 0.05.

## Results

### Participant characteristics

The median age of the participants in both groups was 8 years, and the groups did not differ significantly in age or sex (*p* > 0.05). In the allergy group, the most common diagnosis was allergic rhinitis (*n* = 24), followed by atopic dermatitis (*n* = 15) and allergic asthma (*n* = 7); four children had more than one allergic condition (concurrent rhinitis and asthma). Consistent with the inclusion criteria, none of the allergic patients was diagnosed of allergic conjunctivitis. The demographic characteristics of patients in the study groups are shown in Table 1. There were no significant differences in refractive status or corneal measurements between the allergy and control groups (all *p* > 0.05), confirming that the cohorts were comparable in terms of keratorefractive parameters.

**Table 1:** Baseline demographic and ocular characteristics of children with allergies versus healthy controls.

Ocular surface parameter	Allergy group		Control group		
	Mean ± SD	Min–Max (Median)	Mean ± SD	Min–Max (Median)	*p* ^a^
Age	8.4 ± 2.4	5–14 (8)	8.9 ± 2.7	4–15 (8)	0.339
Sex *n* (%)	Female	23 (46.0)	25 (50.0)	0.69^b^	
	Male	27 (54.0)	25 (50.0)		
Spherical equivalent of refractive status (D)	−0.27 ± 0.7	−2 to 1 (−0.3)	−0.38 ± 0.78	−2.25 to 1.25 (−0.5)	0.555
K1(D)	44.0 ± 2.2	41–50 (43.6)	44.0 ± 2.5	40–51.5 (44)	0.934
K2(D)	44.3 ± 2.4	40.75–49 (44)	43.7 ± 2.1	40–52 (43.5)	0.363
CCT(um)	537.6 ± 27.1	494–604 (536)	534.1 ± 26.7	496–593 (531.5)	0.539
WTW(mm)	11.1 ± 0.5	10.1–11.9 (11.2)	11.0 ± 0.6	9–12.2 (11)	0.704
ACD(mm)	2.95 ± 0.30	2.3–3.6 (3)	2.94 ± 0.31	2–3.98 (2.9)	0.562

CCT, central corneal thickness; WTW, white-to-white corneal distance measurement; ACD, anterior chamber depth. ^a^Mann–Whitney *U* test; ^b^Chi-square test.

### Ocular surface findings

In ocular surface measurements obtained with a corneal topography device, the percentage of meibomian gland atrophy rate was 24.4% ± 16.6% in the allergy group compared to 15.8% ± 12.3% in the control group, and the mean OSDI scores were 12 ± 9 in the allergy group and 8 ± 6 in the control group, which was statistically significant (*p* = 0.008; *p* = 0.044). In parallel with these findings, the duration of NiBUT was significantly lower (8.4 ± 4.0 vs. 10.7 ± 3.4) in the allergic group (*p* = 0.004). There was no significant difference in the limbal redness score or tear meniscus layer thickness between the groups (*p* = 0.06; *p* = 0,71). The differences in the topographic evaluations between the groups are summarized in Table 2. When comparing the allergic subgroups, no single diagnosis (rhinitis, dermatitis, or asthma) was observed to drive ocular changes disproportionately. However, our sample size was not large enough to perform a powered subgroup analysis.

**Table 2:** Ocular surface parameters and topographic measurements in allergic children compared to controls.

Ocular surface parameter		Allergy group		Control group		*p*
Limbal redness score (0–4) *n* (%)	0	22 (44.0)		34 (68.0)		0.060^b^
	1	19 (38.0)		13 (26.0)		
	2	7 (14.0)		3 (6.0)		
	3	2 (4.0)		0 (0.0)		
Ocular surface parameter		Mean ± SD	Min–Max (Median)	Mean ± SD	Min–Max (Median)	*p* ^a^
Meibomian gland atrophy (%)		24.4 ± 16.6	4–66 (22)	15.8 ± 12.3	2–62 (12)	0.008^c^
Tear film layer (um)		188.4 ± 52	100–330 (180)	185.4 ± 54.6	80–380 (175)	0.716
NiBUT (s)		8.44 ± 4.03	2–16 (8)	10.78 ± 3.46	1.8–17 (10.5)	0.004^c^
OSDI Score		12.0 ± 9.4	0–36 (8.5)	8.1 ± 6.2	0–21 (6)	0.044^c^

^a^ Mann‒Whitney *U* test; ^b^Chi-square test; ^c^statistically significant; NiBUT, non-invasive break-up time; OSDI, ocular surface disease index.

### Interobserver agreement

Bland–Altman analysis was used to evaluate the agreement between the two observers’ measurements of the meibomian gland atrophy rate. The mean difference (bias) between observers was +0.7%, indicating that one researcher tended to measure slightly higher atrophy rates, on average. The 95% limits of agreement ranged from −5.8% to +7.2%, suggesting that most differences between observers fell within this range. Notably, 96% of the paired observations were within these limits, demonstrating a high level of agreement and no evidence of systematic bias across measurements.

### Correlation and regression analysis

**Figure 1:**
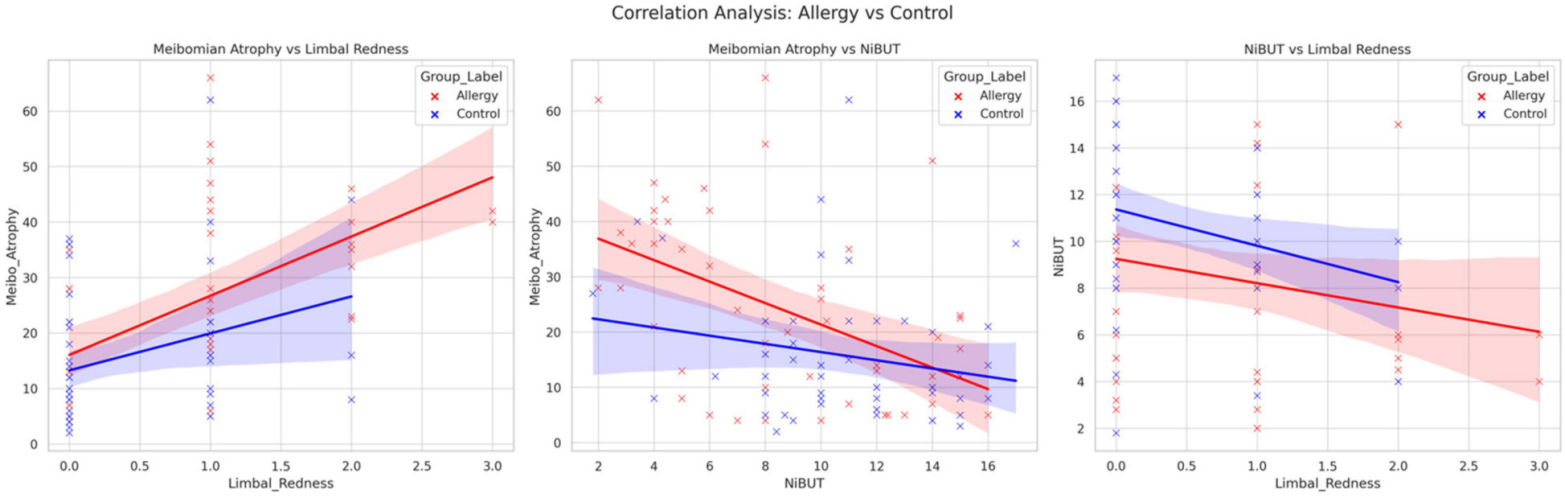
Correlation between meibomian gland atrophy, limbal redness, and Non-invasive tear break-Up time in pediatric allergy and control groups. Scatter plots illustrating the relationships between meibomian gland atrophy (%), limbal redness score, and non-invasive tear break-up time (NiBUT) in children with systemic allergies (red) and healthy controls (blue). The regression lines demonstrate the direction and strength of the associations within each group.

When the relationships between the keratorefractive parameters and ocular surface topographic measurements were analyzed via Spearman's correlation, a significant positive correlation was detected between the meibomian gland atrophy rate and the limbal redness score (*r* = 0.61, *p* < 0.001), and a significant negative correlation with the NiBUT duration (*r* = −0.49, *p* < 0.001) was detected in allergic patients (Figure 1). In the control group, there was a weak but significant correlation between meibomian gland atrophy rate, WTW measurement, and limbal redness score (*r* = 0.29, *p* = 0.037; *r* = 0.32, *p* = 0.023).

In the multivariate linear regression analysis (Table 3) for the allergic group, the limbal redness score (*β* = 0.46; *p* < 0.001) and NiBUT (*β* = −0.36; *p* = 0.005) were identified as significant predictors of the meibomian gland atrophy percentage. However, in the control group, only the limbal redness score was a weak but significant predictor (*β* = 0.31, *p* = 0.041).

**Table 3:** Multivariate linear regression identifying predictors of meibomian gland atrophy in allergic and control groups.

Group	Predictor variable	Unstandardized coefficients	Standardized coefficients	*p*
		B	Beta	
Allergy	Constant	36.749		
	Limbal score	9.064	0.460	˂0.001^a^
	Tear film layer (um)	−0.032	−0.100	0.387
	NiBUT (s)	−1.503	−0.366	0.005^a^
	OSDI score	−0.060	−0.034	0.781
Control	Constant	14.234		
	Limbal score	6.422	0.314	0.041^a^
	Tear film layer (um)	0.020	0.088	0.548
	NiBUT (s)	−0.479	−0.134	0.370
	OSDI score	0.079	0.040	0.784
R square		0.436	0.130	
Dependant variable: meibomian gland atrophy (%)				

^a^ Statistically significant; NiBUT, non-invasive break-up time; OSDI, ocular surface disease index.

## Discussion

The development of atopy and allergic conjunctivitis is rooted in complex immune dysregulation, characterized by an imbalance in the T helper cell response. In atopic individuals, the immune system exhibits a heightened Th2 response, leading to the production of IgE antibodies and the release of inflammatory mediators, such as histamine and leukotrienes, which trigger the characteristic symptoms of atopic dermatitis, asthma, and allergic rhinitis. Similarly, in allergic conjunctivitis, the Th2-dominant immune response drives the production of IgE, mast cell activation, and release of proinflammatory cytokines, ultimately resulting in characteristic ocular signs and symptoms (9, 11). Although respiratory allergies are thought to have a common pathophysiology, the presence of one is not a diagnostic prerequisite for another.

Research indicates that children with allergic conjunctivitis have a higher occurrence of meibomian gland atrophy than their non-allergic counterparts. The inflammatory response linked to allergic conjunctivitis can cause morphological changes in MGs, such as atrophy and tortuosity, which may serve as early signs of MGD (7, 8). Mild ocular involvement in atopic dermatitis has been reported in as many as 40% of cases (12). Atopy itself has also been associated with MGD, tear film instability, goblet cell loss, and conjunctival metaplasia (13).

In our study, we aimed to establish a narrow selection criterion, both clinically and refractively, to exclude patients with missed diagnoses of ocular surface disease. One of the goals of including the OSDI questionnaire was to use a standardized tool to screen for significant ocular surface symptoms while being aware of its limitations in children. As expected, despite a statistically significant difference between the groups (*p* = 0.044), the median OSDI scores were low and clinically similar (9 in the allergy group vs. 6 in the control group). These subthreshold values confirmed that both cohorts were mostly asymptomatic, reinforcing the subclinical nature of our other findings. However, this also emphasizes the difficulty of using symptom scores such as the OSDI to identify early-stage ocular surface disease in children, who may not report symptoms as easily as adults. Additionally, since we wanted to emphasize the importance of subclinical changes, we observed that most patients in both groups clustered around 0 or 1 on the limbal redness scale.

In summary, a statistically significant increase in meibomian gland atrophy was observed in allergic patients who were considered ophthalmologically asymptomatic. Although this level of atrophy falls below the typical threshold for clinical diagnosis and is therefore subclinical, it indicates a measurable difference between groups. Although this increased meibomian gland atrophy was linked to a notably decreased tear break-up time, it may not lead to complaints detectable by the OSDI. These findings highlight that the OSDI may not be sensitive enough to capture the subtle discomfort associated with these subclinical changes, a known limitation when applying adult-oriented questionnaires to pediatric populations.

We included the white-to-white (WTW) diameter to ensure that our wide-ranging age group was homogeneous, given the known correlation between WTW and age (14). Since our study included a wide age range, we deemed it appropriate to include this parameter to assess whether we had obtained a homogeneous group. Additionally, abnormal WTW measurements can occur in the early stages of keratoconus or other ectatic disorders that are often associated with allergic corneal diseases (15). We interpreted the correlation between meibomian atrophy and WTW in healthy controls as a mechanical effect on the eyelids, where a narrower palpebral fissure may impede gland function rather than as an inflammatory change.

Corneal Scheimpflug topography is vital for evaluating children with allergic conjunctivitis, as this common condition can lead to corneal changes, such as astigmatism and keratoconus. The importance of topography in the early detection and management of these issues has been well established (16–20). Frequent eye rubbing and inflammation from recurrent allergic conjunctivitis are major factors contributing to astigmatism, other keratorefractive effects, and overall health of the meibomian glands (3, 21). The presence of significant subclinical findings in our cohort highlighted the need for closer monitoring. Children often do not report MGD symptoms such as irritation, and significant meibomian gland atrophy is prevalent even in asymptomatic cases. This, combined with risk factors such as allergens and screen time, makes routine screening for ocular surface changes essential for all children (22). In a study examining the morphology of the upper and lower eyelids in children and adolescents, the meibomian glands in the pediatric age group did not show significant atrophic changes similar to those in elderly adults. They also reported that meibomian gland dropout did not correlate with age (23). Existing meibomian grading systems are limited by high atrophy thresholds (25%–33%) that would have labeled all our pediatric subjects as Grade 0. This overlooks the significant difference we observed between healthy controls and asymptomatic allergic patients (15.8% vs. 24.4% atrophy), revealing that these seemingly subthreshold values are clinically important (23, 24). In a different study using the LipiView II device, values of 25% or lower (Meiboscore I and 0) were observed in 88% of the normal pediatric population (3). Another study showed significant differences in lower eyelid gland dropout rates between healthy children and those with vernal keratoconjunctivitis; however, the average dropout rates of 20% and 26% were similar, and when these were considered as scores rather than continuous variables, they may not be meaningful (23)..

The main reason why we obtained different results from the literature may be due to several factors. First, our study population was narrowly defined, excluding children with significant screening time, which is a known risk factor for meibomian gland dysfunction. By contrast, Gupta et al. examined a broader population without such specific exclusions (3). Second, demographic and ethnic differences among study populations could also affect baseline variations in meibomian gland morphology. Therefore, we believe that allergic or atopic complaints should be checked during routine check-ups of healthy children from an ophthalmological perspective and that a detailed ocular surface evaluation should be included in at least annual examinations when such complaints or diagnoses are present.

Non-invasive break-up time (NiBUT) is a reliable, objective marker of tear film stability that is ideal for pediatric use because it is non-disruptive and repeatable. Lower NiBUT values are linked to allergic conjunctivitis and dry eye, making them a crucial preventive tool for children who are often asymptomatic until significant ocular surface damage occurs. While obtaining consistent measurements in children is challenging, we ensured accuracy by taking a single cooperative measurement from each participant to avoid bias from repeated testing (25, 26). Additionally, as shown in Figure 1, as limbal redness increases, NiBUT tends to get shorter. This suggests that greater ocular surface inflammation is linked to lower tear film stability in both groups.

The Ocular Surface Disease Index (OSDI) questionnaire is a recognized tool for assessing dry eye disease (DED) symptoms and their impact on quality of life. Several studies have demonstrated the feasibility of using the OSDI in pediatric populations. Alnahdi et al. used the OSDI questionnaire to assess dry eye symptoms in children aged 1–18 years, confirming its applicability (27). Furthermore, the OSDI has been reported to be a valid instrument for assessing dry eye symptoms in children undergoing various ophthalmic treatments, indicating its versatility across different clinical contexts (28–31). However, it has also been noted that children may report fewer symptoms than adults, which could lead to an underestimation of the severity of dry eye conditions when OSDI is used (30, 32).

This study had several limitations. The cross-sectional design prevents causal conclusions, and the lack of follow-up data limits our understanding of the progression of these findings. The generalizability of our results may be restricted by strict exclusion criteria and the use of the adult OSDI questionnaire in the pediatric population. Furthermore, a potential ‘healthy user’ bias in our control group may have amplified the observed differences. Finally, although most participants were assessed before starting treatment, the unrecorded use of over-the-counter allergy medications is a potential confounder.

This study demonstrates that systemic atopy in children, even without ocular symptoms, is associated with significant subclinical ocular surface changes, including increased meibomian gland atrophy and reduced tear film stability. This suggests that systemic atopic processes may influence the ocular surface before obvious symptoms of allergic conjunctivitis develop. Therefore, even without ocular symptoms, allergic children exhibit measurable differences in ocular surface health. Although the differences in Ocular Surface Disease Index (OSDI) scores were statistically significant, the scores for both groups were below thresholds indicating clinical severity, highlighting the limitations of symptom-based assessments in children. While the use of corneal topography and gland imaging in asymptomatic children remains exploratory, previous studies demonstrating early ocular changes in atopic groups support their potential application. However, these minor changes do not alter daily clinical practice. Long-term studies are necessary to determine their prognostic value and to guide future screening strategies before these techniques become routine.

## Data Availability

The raw data supporting the conclusions of this article will be made available by the authors, without undue reservation.
